# Psychosis and Seizures Attributed to Severe Vitamin B12 Deficiency: A Case Report

**DOI:** 10.7759/cureus.39889

**Published:** 2023-06-02

**Authors:** Shellsea Portillo, Nehemias A Guevara, Garry Francis-Morel

**Affiliations:** 1 Internal Medicine, St. Barnabas Hospital Health System, Bronx, USA; 2 Internal Medicine, St. Barnabas Medical Center, Bronx, USA

**Keywords:** vitamin b12, psychosis, seizures, paresthesias, vitamin b12 deficiency anemia

## Abstract

Vitamin B12 deficiency is known to cause a variety of symptoms, including megaloblastic anemia, glossitis, and neuropsychiatric disorders. This case report describes a patient who presented with cognitive decline, psychosis, and seizures due to a severe vitamin B12 deficiency. Following treatment with vitamin supplementation therapy, the patient's condition significantly improved. The literature has also documented similar neuropsychiatric manifestations of vitamin B12 deficiency, highlighting the potential for symptom reversal with prompt and appropriate treatment. Therefore, early diagnosis and treatment of vitamin B12 deficiency are critical to preventing potentially irreversible neurological damage.

## Introduction

Vitamin B12 deficiency is a prevalent condition often accompanied by neurological and behavioral symptoms, including depression, irritability, cognitive decline, impaired memory, dementia, psychosis, delirium, subacute combined degeneration, and peripheral sensory neuropathies. Additionally, epileptic seizures have been reported as a potential manifestation of this deficiency [1-5]. Although the effects of vitamin B12 deficiency on hematological disorders have been extensively studied, neuropsychiatric symptoms are frequently overlooked or misdiagnosed. Neuropsychiatric symptoms may occur in individuals with vitamin B12 insufficiency, even without anemia or macrocytosis [6]. In one study, 28% of 141 consecutive patients with neuropsychiatric symptoms had normal hematocrit, mean corpuscular volume, or both [7]. Failure to recognize the absence of conventional hematological and neurological symptoms can delay diagnosis, which can have serious consequences given that neuronal damage may become irreversible if therapy is delayed [8]. Despite the known neuropsychiatric implications of B12 deficiency, the underlying mechanisms remain unclear. The proposed mechanisms include changes in one-carbon metabolism, dysregulation of folate metabolism, and hereditary susceptibility [9].

This paper describes a patient who presented with gradual cognitive and functional decline, acute psychosis, and seizures, which were ultimately attributed to pernicious anemia. Nevertheless, the patient recovered remarkably following vitamin supplementation. Similar presentations of vitamin B12 deficiency have been described in the literature, underscoring the need for early diagnosis and therapy to prevent irreversible neuronal damage.

## Case presentation

**Figure 1:**
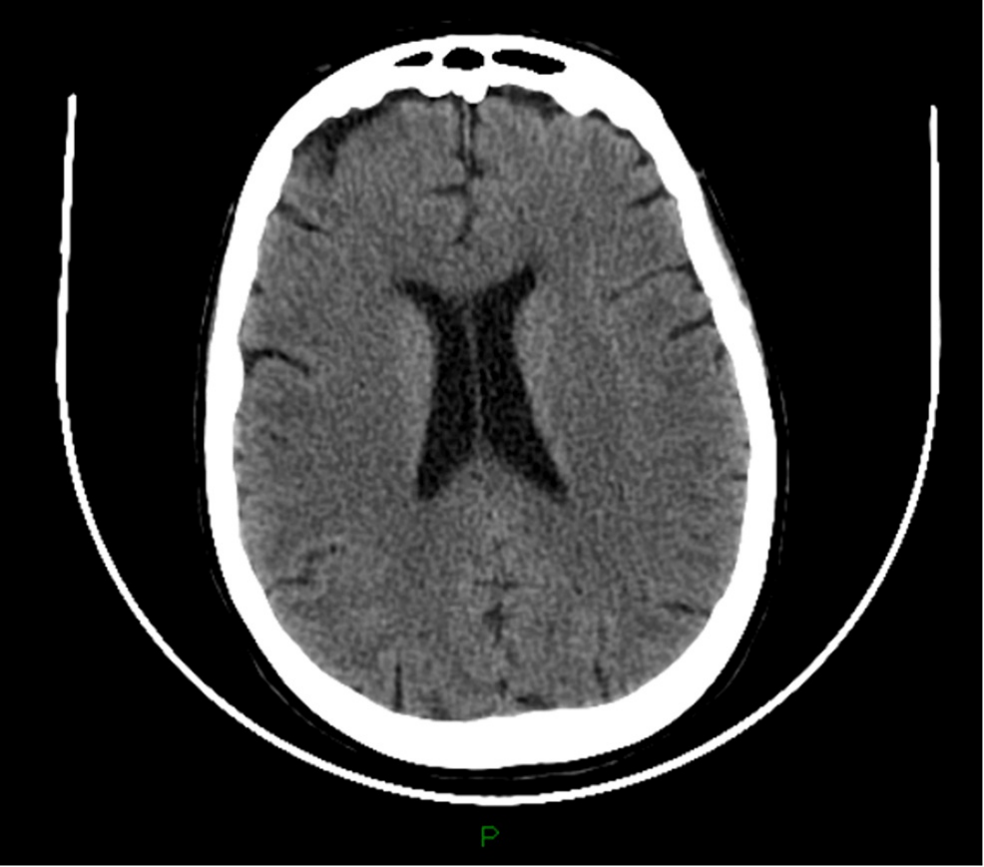
CT head No intra- or extra-axial hemorrhage. No evidence of acute large vessel infarct. No intracranial mass effect. There is preservation of the gray/white matter interface.

A 61-year-old woman with no medical conditions other than a history of frequent falls was brought to the emergency department (ED) by emergency medical services (EMS) due to erratic behavior. She was found inappropriately dressed, crawling on the ground on a sidewalk near a bus stop. She had reported intermittent epigastric and periumbilical pain, bilateral lower-extremity muscle spasms, paresis, and paresthesia that were exacerbated by long-distance walking. Upon admission, her vital signs were: temperature, 98.9 °F (37.1 °C); blood pressure, 115/73 mm Hg; heart rate, 89; respiratory rate, 16 breaths/min; oxygen saturation, 98% on room air. Physical examination revealed an unsteady gait, increased deep tendon reflexes in the bilateral lower extremities, and lethargy with a Glasgow Coma Scale (GCS) of 13. Laboratory workup revealed macrocytic anemia with a hemoglobin of 8.1 g/dL, mild thrombocytopenia, severe vitamin B12 deficiency of 0 pg/mL, positive anti-intrinsic factor, and anti-parietal cell antibodies, as shown in Table 1. She was diagnosed with pernicious anemia, and her altered mental status was attributed to a severe vitamin B12 deficiency. During admission, the patient experienced a single tonic-clonic seizure episode. The inpatient electroencephalogram (EEG) was abnormal due to the slowing of the background and excess diffuse slowing. As per neurology's interpretation, these findings in cerebral activity could be postictal in nature since the EEG was recorded within a short time after a seizure. Head computerized tomography (CT) showed no acute intracranial findings, as shown in Figure 1. Brain MRI with and without contrast showed no evidence of an infarct, hemorrhage, or focal mass lesion. No abnormal enhancement or pathologically enhancing lesions on postcontrast imaging were identified. Treatment with 1000 µg of cyanocobalamin IM daily was initiated, resulting in a noticeable improvement of lower extremity paresis and paresthesia as well as increased vitamin B12 levels. She was discharged with instructions to continue vitamin B12 treatment (1000 mcg IM once per month) and referrals for evaluations by hematology, neurology, physical therapy, and behavioral health specialists. The ambulatory awake EEG results two months later were normal. The EEG report was the only one available since electroencephalogram data was lost a month before this admission. Antiepileptic medications were not prescribed since the patient did not have any personal or family history of seizures. During her outpatient follow-up, the patient reported a complete resolution of her neuropsychiatric symptoms and denied any other episodes of seizures.

**Table 1:** Laboratory analyses MCV: mean corpuscular volume; MCH: mean corpuscular hemoglobin; MCHC: mean corpuscular hemoglobin concentration; RDW: red cell distribution width, RBCs: red blood cells. The presence of intrinsic factor antibodies and anti-parietal cell antibodies as well as megaloblastic anemia and low serum vitamin B12 are diagnostic of pernicious anemia. MCV is a measurement of the average size of the RBCs; MCH reflects the average amount of hemoglobin in the RBCs; MCHC is the ratio of the MCH to the MCV; RDW reflects the degree of variation in size of the RBCs. These are usually abnormal in various types of anemia including pernicious anemia.

Parameter	Initial values	Repeated values	Units	Reference values
Hemoglobin	8.1	13.9	g/dL	11.2–15.7 g/dL
Platelets	123	298	10^3^/µL	150–450 × 10^3^/µL
MCV	131.4	107.3	fL	79.4–94.8 fL
MCH	47.9	34.8	pg	25.6–32.2 pg
MCHC	36.5	32.4	g/dL	32.2–35.5 g/dL
RDW	14.5	13.2	%	11.7–14.4 %
Vitamin B12	0	912	pg/mL	160–950 pg/mL
Antiparietal cell antibody	43.5		Units	0.0–20.0 Units
Intrinsic factor antibody	88.7		AU/mL	0.0–1.1 AU/mL

## Discussion

The combination of progressive cognitive and functional decline, psychosis, and seizures is a rare presentation of vitamin B12 deficiency [1-3,5]. However, most case reports have described complete symptom remission with vitamin replacement therapy, even after the discontinuation of antipsychotic and antiepileptic medications. Unfortunately, some others endured long-term damage. For example, a patient described by Silva et al. had persistent psychotic symptoms and seizures despite cyanocobalamin replacement therapy after discontinuing antipsychotic and antiepileptic medications [5]. Delay in diagnosis may have played a role because neurons with destroyed myelin sheaths due to long-term vitamin B12 deficiency are more susceptible to the excitotoxic effects of glutamate, leading to irreversible axonal damage associated with psychosis and epilepsy [10]. Despite the enduring neuropsychiatric effects, cognitive and functional recovery is still possible with vitamin B12 supplementation.

Vitamin B12 deficiency should be included in the differential diagnosis of patients presenting with atypical psychosis or seizures, particularly when cognitive decline occurs [1-3,5]. Serum B12 levels have limited sensitivity and specificity, and clinicians should be aware that concurrent antiepileptic medication and sample exposure to room temperature for over two hours may result in false results. Hence, serum methylmalonic acid levels (MMA) and total homocysteine should be measured in cases where cobalamin insufficiency is suspected but classic symptoms are absent and serum B12 levels are normal or low normal and since both proved to be highly sensitive tests of deficiency [11-13].

The current study was based on a single case report, which limits the applicability of the findings to a larger population. Our patient's seizure episode and initial EEG results were consistent with seizure activity. However, subsequent EEG findings normalized after B12 replacement therapy, demonstrating that B12 deficiency-related neurologic disorders can be reversed with vitamin replacement therapy, sometimes even after a prolonged deficit status. This case highlights the importance of early diagnosis and treatment of B12 deficiency, especially in patients with neuropsychiatric symptoms such as seizures and psychosis. Diagnosing B12 insufficiency in patients presenting to the emergency department with unusual behavior is challenging. It is crucial to differentiate between reversible and potentially treatable causes of aberrant behavior. Nevertheless, this case report provides essential insights into the potential neuropsychiatric effects of severe vitamin B12 deficiency, which can encourage additional research in this field. Further research is necessary to understand the pathogenesis and long-term effects of B12 deficiency-induced neuropsychiatric disorders.

## Conclusions

In conclusion, this case report underscores the significance of administering screening tests for vitamin B12 deficiencies among individuals who present to the emergency department with neuropsychiatric symptoms, particularly in the absence of prior neurological or psychiatric conditions. The findings suggest that such evaluations may be critical for the early identification and intervention of vitamin B12 deficiencies in patients with neuropsychiatric symptoms. Further research is needed to determine the optimal screening strategies and diagnostic criteria for detecting vitamin B12 deficiencies in the emergency department setting.
